# Soy Isoflavone Genistein Inhibits an Axillary Osmidrosis Risk Factor ABCC11: In Vitro Screening and Fractional Approach for ABCC11-Inhibitory Activities in Plant Extracts and Dietary Flavonoids

**DOI:** 10.3390/nu12082452

**Published:** 2020-08-14

**Authors:** Hiroki Saito, Yu Toyoda, Hiroshi Hirata, Ami Ota-Kontani, Youichi Tsuchiya, Tappei Takada, Hiroshi Suzuki

**Affiliations:** 1 Frontier Laboratories for Value Creation, Sapporo Holdings Ltd., 10 Okatome, Yaizu, Shizuoka 425-0013, Japan; hiroki.saito@sapporoholdings.co.jp (H.S.); hiroshi.hirata@sapporoholdings.co.jp (H.H.); ami.ota@sapporoholdings.co.jp (A.O.-K.); yoichi.tsuchiya@sapporoholdings.co.jp (Y.T.); 2 Department of Pharmacy, The University of Tokyo Hospital, 7-3-1 Hongo, Bunkyo-ku, Tokyo 113-8655, Japan; tappei-tky@umin.ac.jp (T.T.); suzukihi-tky@umin.ac.jp (H.S.)

**Keywords:** axillary osmidrosis treatment, bioactivity investigation of food extract, body odor, food ingredient, functional food, *Glycine max*, health promotion, MRP8, phytochemicals, transporter

## Abstract

Axillary osmidrosis (AO) is a common chronic skin condition characterized by unpleasant body odors emanating from the armpits, and its aetiology is not fully understood. AO can seriously impair the psychosocial well-being of the affected individuals; however, no causal therapy has been established for it other than surgical treatment. Recent studies have revealed that human ATP-binding cassette transporter C11 (ABCC11) is an AO risk factor when it is expressed in the axillary apocrine glands—the sources of the offensive odors. Hence, identifying safe ways to inhibit ABCC11 may offer a breakthrough in treating AO. We herein screened for ABCC11-inhibitory activities in 34 natural products derived from plants cultivated for human consumption using an in vitro assay system to measure the ABCC11-mediated transport of radiolabeled dehydroepiandrosterone sulfate (DHEA-S—an ABCC11 substrate). The water extract of soybean (*Glycine max*) was found to exhibit the strongest transport inhibition. From this extract, via a fractionation approach, we successfully isolated and identified genistein, a soy isoflavone, as a novel ABCC11 inhibitor with a half-maximal inhibitory concentration value of 61.5 μM. Furthermore, we examined the effects of other dietary flavonoids on the ABCC11-mediated DHEA-S transport to uncover the effects of these phytochemicals on ABCC11 function. While further human studies are needed, our findings here about the natural compounds will help develop a non-surgical therapy for AO.

## 1. Introduction

Offensive or strong body odors can be a source of social embarrassment. Axillary osmidrosis (AO) is a chronic skin condition characterized by such body odors and excessive sweating from the armpits [1]. In Asian countries such as Japan and China where fewer people have strong body odor, AO is perceived even more negatively [2]. However, except for surgical treatments, no causal therapy has been established for AO.

The inhibition of human ATP-binding cassette transporter C11 (ABCC11, also known as MRP8)—a risk factor of AO—may induce physiological changes related to body odors [1,3,4,5]. ABCC11 is one of the ABC proteins that transport various molecules across cellular membranes in an ATP-dependent manner [6,7]. A non-synonymous single nucleotide polymorphism c.538G>A (p.Gly180Arg) in the *ABCC11* gene, which codes a functionally null variant with a high allele frequency in East Asians [8], has been found to be a determinant of AO risk [1,3,4,5]. Considering the facts that (1) genetically *ABCC11*-deficient subjects carry little AO risk and (2) the ABCC11 wild-type (WT) is expressed in human axillary apocrine glands that produce a variety of odor precursors [9], the inhibition of ABCC11 may lead to ways to prevent and treat AO. However, no medication is currently approved for AO treatment by ABCC11 inhibition. Hence, the exploration and identification of biologically safe ABCC11 inhibitors is an important issue.

In this study, we examined the ABCC11-inhibitory activities of 34 dietary plant products using an in vitro transport assay system. By screening the plant extracts and a subsequent fractional approach, genistein, a well-recognized soy isoflavone, was identified as a novel ABCC11 inhibitor with a half-maximal inhibitory concentration (IC_50_) of 61.5 μM. Moreover, since little is known about food ingredients with the potential to inhibit ABCC11, we further investigated the effects of other dietary flavonoids on the ABCC11 function.

## 2. Materials and Methods

### 2.1. Materials

The key materials and resources used in this study are summarized in Table 1. All other chemicals used were commercially available and of analytical grade. The full-length human ABCC11 WT (NCBI accession: NM_033151) open reading frame in pcDNA3.1/hygro(−) plasmid [4] and recombinant adenoviruses for the expression of the human ABCC11 WT [10] were constructed in our previous studies; the plasmid/adenovirus vectors and the corresponding control vectors were prepared as a new experimental lot in this study. The plant materials (Table A1) were purchased from local supermarkets in Shizuoka, Japan, between July 2016 and July 2017.

### 2.2. Preparation of Plant Extracts

After the fruits were cleaned, the peels and pulps were carefully separated. The fresh and dried materials (summarized in Table A1) were finely chopped with a knife and ground using a mill (Crush Millser IFM-C20G; Iwatani, Tokyo, Japan), respectively. In the subsequent extraction step, approximately 50 g of the preprocessed plant material were well liquidized in 100 mL of distilled water using a juicer (Crush Millser IFM-C20G; Iwatani) and stirred for 30 min at room temperature. The suspension was centrifuged at 12,000× *g* at 4 °C for 10 min to remove the debris. The supernatant was collected and passed through ordinary filter paper. The filtrate was dialyzed against distilled water (500 mL) at 4 °C overnight with a dialysis membrane with a molecular weight cut-off of 14,000 (Spectrum Chemical Mfg, New Brunswick, NY, USA). The distilled water containing the small molecules that passed the dialysis membrane was lyophilized using FDU-2000 (EYELA, Tokyo, Japan). The freeze-dried extracts were stored at −20 °C, dissolved in ultrapure water at 10 mg/mL (10,000 ppm), and subjected to sonication as appropriate before use. Then, 5 μL of the solution were mixed with 20 μL of a transport buffer (10 mM Tris/HCl, 250 mM sucrose, and 10 mM MgCl_2_, and pH 7.4); 1 μL of this clear liquid was used for a vesicle transport assay (total 20 μL/sample), as described below.

### 2.3. Cell Culture

Human embryonic kidney 293 (HEK293)-derived 293A cells were maintained in Dulbecco’s Modified Eagle’s Medium (Nacalai Tesque, Kyoto, Japan) supplemented with 10% fetal bovine serum (Biowest, Nuaillé, France), 1% penicillin-streptomycin (Nacalai Tesque), 2 mM L-glutamine (Nacalai Tesque), and 1 × non-essential amino acid (Life Technologies, Tokyo, Japan) at 37 °C in a humidified atmosphere of 5% CO_2_ in air (*v*/*v*), following our previous study [11]. To obtain ABCC11-expressing 293A cells for the plasma membrane vesicles, we performed plasmid transfection using polyethylenimine MAX (1 mg/mL in Milli-Q water, pH 7.0; Polysciences, Warrington, PA, USA) [12] or adenovirus infection [9], as described previously.

### 2.4. Preparation of ABCC11-Expressing Plasma Membrane Vesicles

Plasma membrane vesicles were prepared from ABCC11-expressing 293A cells or control cells, as described previously [12], and then rapidly frozen in liquid N_2_ and stored at −80 °C until use. Unless otherwise indicated, the plasma membrane vesicles used in the present study were derived from 293A cells 48 h after the plasmid transfection. The protein concentration of the plasma membrane vesicles was quantified using a BCA Protein Assay Kit (Pierce, Rockford, IL, USA) with bovine serum albumin as a standard according to the manufacturer’s protocol.

### 2.5. Immunoblotting

The expression of ABCC11 protein in plasma membrane vesicles was examined by immunoblotting, as described previously [4,9] with minor modifications. Briefly, the prepared samples were electrophoretically separated on poly-acrylamide gels and transferred to a Hybond^®^ ECL^TM^ nitrocellulose membrane (GE Healthcare, Buckinghamshire, UK) by electroblotting at 15 V for 70 min. After blocking by Tris-buffered saline containing 0.05% Tween 20 and 5% skim milk (TBST-skim milk) at 4 °C overnight, blots on the membrane were probed with a rat monoclonal anti-ABCC11 antibody (M8I-74; Abcam, Cambridge, MA, USA; diluted 200 fold) and a rabbit polyclonal anti-Na^+^/K^+^-ATPase α antibody (sc-28800; Santa Cruz Biotechnology, Santa Cruz, CA, USA; diluted 1000 fold), followed by incubation with a goat anti-rat immunoglobulin G (IgG)–horseradish peroxidase (HRP) conjugated antibody (NA935V; GE Healthcare; diluted 2000 fold) and a donkey anti-rabbit IgG–HRP conjugated antibody (NA934V; GE Healthcare; diluted 3000 fold), respectively. All antibodies were used in TBST-skim milk. HRP-dependent luminescence was developed using the ECL^TM^ Prime Western Blotting Detection Reagent (GE Healthcare) and detected using a multi-imaging Analyzer Fusion Solo 4^TM^ system (Vilber Lourmat, Eberhardzell, Germany).

### 2.6. Vesicle Transport Assay

The inhibitory effects of the various target extracts and compounds on the ABCC11 function were examined using the in vitro vesicle transport assay, a well-established method to quantitatively evaluate ABC transporter function [13]. For this purpose, the ATP-dependent transport of [1,2,6,7-^3^H(N)]-dehydroepiandrosterone sulfate (DHEA-S) (PerkinElmer, Waltham, MA, USA), which is an ABCC11 substrate [7], into the ABCC11-expressing and control plasma membrane vesicles was quantified following our previous study [9] with some minor modifications in the rapid filtration technique, as described below.

In brief, the plasma membrane vesicles (0.25 mg/mL or indicated concentrations) were incubated with [1,2,6,7-^3^H(N)]-DHEA-S (100 nM or indicated concentrations) in a reaction mixture (total 20 μL: 10 mM Tris/HCl, 250 mM sucrose, 10 mM MgCl_2_, 10 mM creatine phosphate, 1 mg/mL creatine phosphokinase, 50 mM ATP or AMP as a substitute of ATP, and pH 7.4) for 5 min at 37 °C, either without (i.e., with only vehicle control) or with the individual target fractions/authentic chemicals at the indicated concentrations. As the vehicle control, 1% water was used for plant extracts; 1% methanol (Nacalai Tesque) or 1% dimethyl sulfoxide (DMSO; Nacalai Tesque) was used for the individual target fractions, as described below. Since stock solutions of authentic chemicals were prepared with DMSO at 10 mM, 1% DMSO was employed as the vehicle control for them. After incubation, the reaction mixture was mixed with 980 μL of an ice-cold stop buffer (2 mM EDTA, 0.25 M sucrose, 0.1 M NaCl, 10 mM Tris-HCl, and pH 7.4) and rapidly filtered on a membrane filter (MF-Millipore Membrane (HAWP02500; Millipore, Tokyo, Japan) for extract screening or Whatman™ Grade GF/F Glass Microfiber Filter Paper (GE Healthcare) for the other experiments). After washing with 5 mL of the ice-cold stop buffer three times, the plasma membrane vesicles trapped on the membrane filter were dissolved in Clear-sol II (Nacalai Tesque). Then, the radioactivity incorporated into the plasma membrane vesicles was measured with a liquid scintillator (Tri-Carb 3110TR; PerkinElmer).

The transport activity in each group was calculated as the incorporated clearance (μL/mg protein/min = incorporated level of DHEA-S (disintegrations per minute (DPM)/mg protein/min)/DHEA-S level in the incubation mixture (DPM/μL)). ATP-dependent DHEA-S transport was calculated by the difference in transport activity with and without ATP. Similarly, ABCC11-mediated DHEA-S transport activity was calculated by subtracting the ATP-dependent DHEA-S transport activity of control plasma membrane vesicles from that of ABCC11-expressing ones. Unless otherwise indicated, effects of the target fractions/compounds on the ATP-dependent DHEA-S transport activity were also examined for the control plasma membrane vesicles.

### 2.7. Fractionation of Soybean (Glycine max) Extract

Medium-pressure liquid chromatography (MPLC) was conducted using a dual channel automated flash chromatography system (EPCLC-W-Prep 2XY; YAMAZEN, Osaka, Japan), as described below. All the eluates were evaporated to dryness and then stored at −20 °C. They were reconstituted in an appropriate solvent before use in the vesicle transport assay for the evaluation of ABCC11-inhibitory activities and/or chemical characterization by mass spectrometry (MS) analysis.

The water extract of dry soybeans was separated into 12 fractions (Fr.#1-12) by MPLC on an octadecyl-silica (ODS) column (DispoPackAT ODS-25; particle size 25 μm, column size 120 g, i.d. 40 × 188 mm; YMC, Kyoto, Japan). The separation was performed in the linear gradient elution mode with solvent A (0.2% formic acid in water) and solvent B (0.2% formic acid in acetonitrile) (solvent A:solvent B (*v*/*v*): 0–5 min 95:5; 5–25 min 95:5 to 0:100; and 25–35 min 0:100) at a flow rate of 40 mL/min, with UV monitoring at 265 nm using an equipped UV detector. Each fraction was reconstituted (10 mg/mL) in an appropriate solvent (i.e., water for Fr.#1 and Fr.#2, 50% methanol for Fr.#3-11, and methanol for Fr.#12) before use.

Among the 12 fractions, Fr.#11 (the target fraction reconstituted in 50% methanol) was further subjected to MPLC over an ODS column (RediSep ODS GOLD; 5.5 g media, 20–40 μm spherical; Teledyne Isco, Lincoln, NE, USA) in the stepwise elution mode using a mixture of the same A and B solvents (solvent A:solvent B (*v*/*v*): 0–2 min 80:20; 2–9 min 50:50; and 9–17 min 0:100) at a flow rate of 15 mL/min with UV monitoring at 254 nm. This gives three subfractions (Fr.#11-1 to Fr.#11-3) plus a dominant peak eluted from 3.0 to 5.2 min. The dominant peak was collected and then further separated in the same column with a linear gradient of 10–50% of solvent B in solvent A to give three more subfractions (Fr.#11-4 to Fr.#11-6).

Finally, to further separate ABCC11-inhibitory ingredients, Fr.#11-5—the most active subfraction among Fr.#11-1 to Fr.#11-6 in terms of ABCC11 inhibition—was purified by a recycling preparative HPLC system (LaboACE LC-5060; Japan Analytical Industry, Tokyo, Japan) equipped with a gel permeation column (JAIGEL-GS310; i.d. 20 × 500 mm; Japan Analytical Industry), using methanol as a mobile phase at 5 mL/min and with refractive index monitoring and UV monitoring at 254 nm. In brief, Fr.#11-5 was separated by the recycling mode for 120 min. Then, Fr.#11-5-1 and Fr.#11-5-2 were collected from 123 to 126 min and from 160 to 176 min, respectively. All the wastes were collected and further processed as Fr.#11-5-3. Additionally, all the subfractions were evaporated to dryness and then stored at −20 °C. They were reconstituted in DMSO (2 mg/mL) before use.

### 2.8. Chemical Characterizations

For the qualitative determination of the isolated compounds, chromatographic separations, and subsequent MS (or MS/MS) analyses were carried out with an LC-quadrupole time-of-flight (Q-TOF)-MS/MS system consisting of an HPLC instrument (Agilent 1100 Series equipped with a diode array and multiple wavelength detector (DAD) (G1316A); Agilent Technologies, Santa Clara, CA, USA) coupled with an Agilent 6510 Q-TOF (Agilent Technologies). The chromatographic conditions and MS setting were drawn from our previous study [14] with some minor modifications. Briefly, the separation was performed on a Zorbax Eclipse Plus C18 column (2.1 × 100 mm; Agilent Technologies) maintained at 40 °C under gradient mobile conditions with a mixture of solvent C (0.1% formic acid in water) and solvent D (acetonitrile) (solvent C:solvent D (*v*/*v*): 0–8 min 95:5 to 5:95, and 8–12 min 5:95) with a flow rate of 0.5 mL/min. The detection range of the DAD was set from 190 to 400 nm, and the MS detection system operated in the positive ionization mode at an MS scan range of *m*/*z* 100–1700. Peak analysis was performed using the Agilent MassHunter Workstation software (version B.03.01; Agilent Technologies).

### 2.9. Calculation of the Half-Maximal Inhibitory Concentration Values

To calculate the IC_50_ value of genistein against DHEA-S transport by ABCC11, the DHEA-S transport activities were measured in the presence of genistein at several concentrations. The ABCC11-mediated DHEA-S transport activities were expressed as a percentage of the control (100%). Based on the calculated values, fitting was carried out with the following formula using the least-squares methods in Excel 2019 (Microsoft, Redmond, WA, USA), as described previously [15]:

(1) Predicted value [%]=100−(Emax×CnEC50n+Cn)

where E_max_ is the maximum effect, EC_50_ is the half maximal effective concentration, C is the concentration of the test compound, and n is the sigmoid-fit factor. IC_50_ was calculated based on these results.

### 2.10. Statistical Analysis

All statistical analyses were performed using Excel 2019 with the Statcel4 add-in software (OMS publishing, Saitama, Japan). Various statistical tests were used for different experiments, as described in the figure legends. Briefly, when analyzing multiple groups, the similarity of variance between groups was compared using Bartlett’s test. When passing the test for homogeneity of variance, a parametric Tukey–Kramer multiple-comparison test or a Dunnett’s test for comparisons with a control group was used. To investigate the inhibitory effect of each dietary food ingredient on ABCC11 function (vs. vehicle control as 100%), one-sample *t*-test (one-sided) was conducted. Statistical significance was defined in terms of *p* < 0.05 or 0.01. The sample sizes were empirically determined to ensure informative results and sufficient material for subsequent studies, and no specific statistical test was used in deciding them. All experiments were monitored in a non-blinded fashion.

### 2.11. Availability of Data and Material

Data supporting the results of this study are included in this published article and its appendix or are available from the corresponding author on reasonable request.

## 3. Results

### 3.1. Confirmation of ABCC11-Mediated Transport Activity

**Figure 1:**
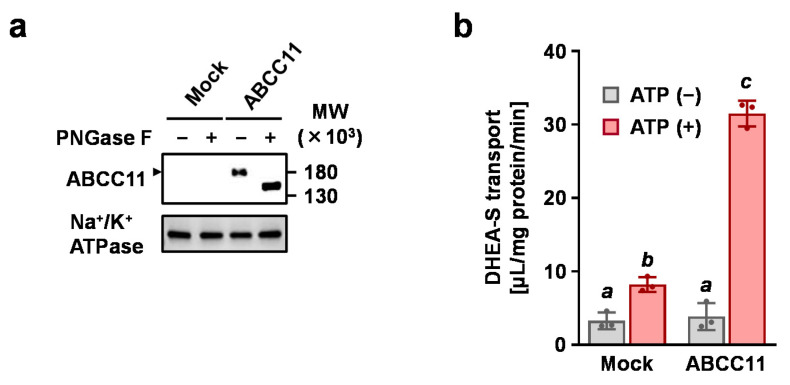
Expression and function of ABCC11. (**a**) Immunoblot detection of ABCC11 protein in the plasma membrane vesicles using an anti-ABCC11 antibody. Mock means plasma membrane vesicles that were prepared from control cells transfected with an empty pcDNA3.1/hyg(−) vector. Arrowhead: matured ABCC11 as an *N*-linked glycosylated protein. Na^+^/K^+^-ATPase (a plasma membrane protein) was used for a loading control. (**b**) [1,2,6,7-^3^H(N)]-dehydroepiandrosterone sulfate (DHEA-S) transport activities. Plasma membrane vesicles were incubated with or without ATP for 5 min. In this assay, all incubation mixtures contained 1% dimethyl sulfoxide (DMSO). Data are expressed as the mean ± SD; *n* = 3. Statistical analyses for significant differences were performed using Bartlett’s test, followed by a parametric Tukey–Kramer multiple-comparison test. Different letters indicate significant differences between groups (*p* < 0.05).

Prior to screening the ABCC11-inhibitory activities of natural products, we verified the transport assay system used in the present study. Immunoblotting with the anti-ABCC11 antibody confirmed the expression of ABCC11 protein as a matured *N*-linked glycoprotein in the plasma membrane vesicles prepared from the ABCC11-expressing cells (Figure 1a). No detectable expression of ABCC11 was observed in the control vesicles. We then measured the ATP-dependent DHEA-S transport into the ABCC11-expressing plasma membrane vesicles (Figure 1b). The DHEA-S transport activities of the ABCC11 vesicles were remarkably higher than those of the mock vesicles, which was enough for the quantitative evaluation of ABCC11-mediated DHEA-S transport activity in subsequent processes.

### 3.2. Screening the ABCC11-Inhibitory Activities of Plant Extracts

**Figure 2:**
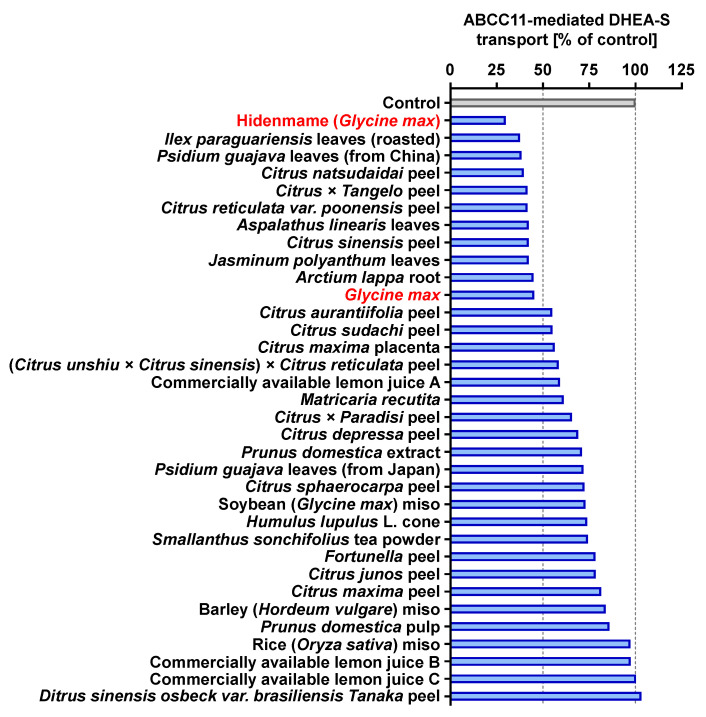
Screening of inhibitory effects of various plant extracts on the transport activity of ABCC11. Inhibitory effect of each plant extract on the ABCC11-mediated [1,2,6,7-^3^H(N)]-DHEA-S transport activity was investigated by the vesicle transport assay. Plasma membrane vesicles (0.375 mg/mL in the reaction mixture) were incubated with the extract (100 ppm) in the presence of 50 μM [1,2,6,7-^3^H(N)]-DHEA-S for 5 min; 1% water was used for the vehicle control. Data are expressed as % of vehicle control, and they represent averages of two independent experiments.

For the ABCC11-inhibitory properties of natural products, we focused on plants commonly found in the human diet including citruses, tea leaves, soybeans, and miso, a traditional grain-based fermented food in Japan [16]. Each sample was extracted with water and then dialyzed, and the resulting outer layer was lyophilized and reconstituted in water at 10 mg/mL. The 34 obtained concentrates (final concentration at 100 ppm) were used for screening the ABCC11-inhibitory activity (Figure 2). Since the extract of soybean (*Glycine max*) showed the highest inhibitory activities (approximately 70% inhibition) and soybean is a common crop consumed globally, we further explored the ingredients therein responsible for the ABCC11-inhibitory activity.

### 3.3. Fractionation and Isolation of Glycine Max (Soybean) Extract by Chromatographic Separations

**Figure 3:**
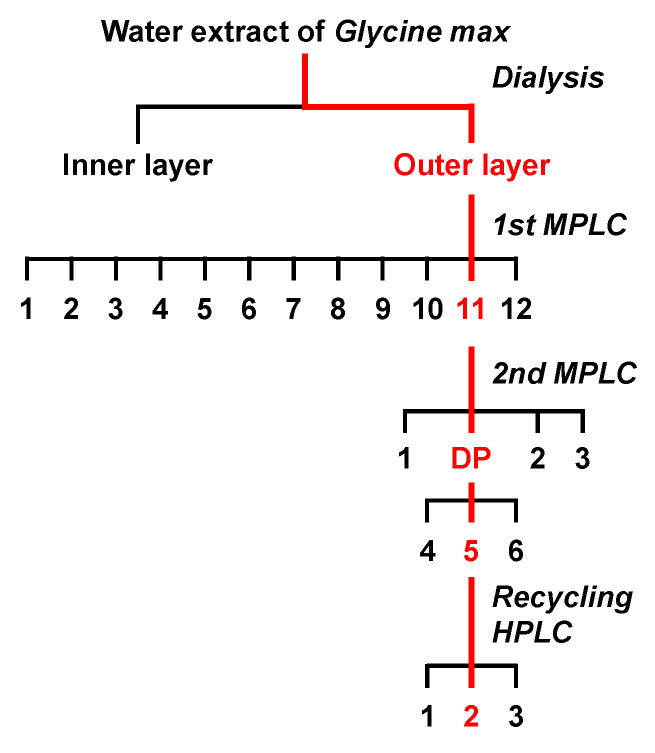
Separation scheme used to fractionate ABCC11 inhibitors in the soybean extract. In each separation step, the fraction with the highest ABCC11-inhibitory activity is colored in red. DP: dominant peak (details are described in Materials and Methods).

**Figure 4:**
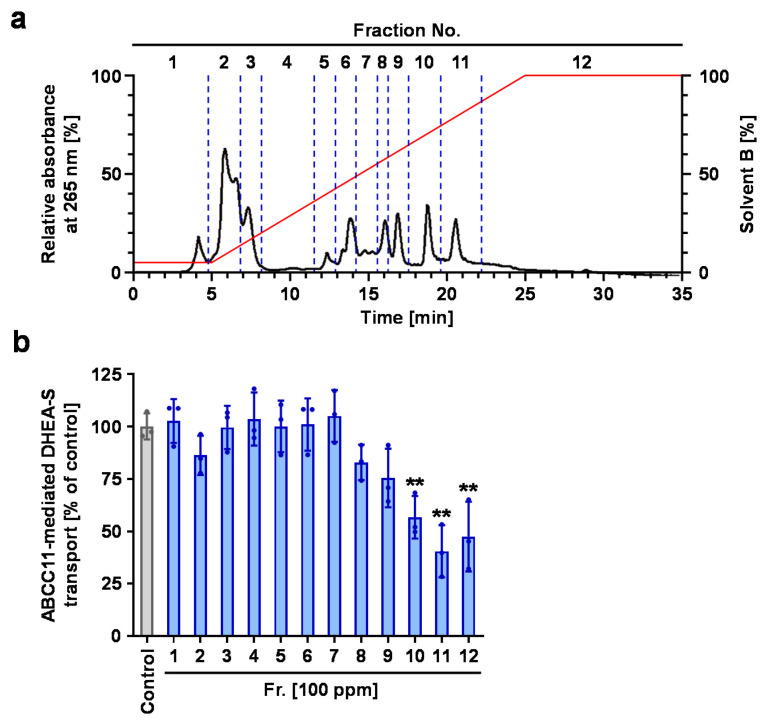
ABCC11-inhibitory activities for each fraction of soybean extract from the first separation step with preparative medium-pressure liquid chromatography (MPLC). (**a**) A preparative MPLC chromatogram for separating the water extract of soybeans. The chromatogram was recorded at 265 nm. Red line indicates linear gradients of solvent B (0.2% formic acid in acetonitrile). (**b**) ABCC11-inhibitory activity profile of each fraction (100 ppm) obtained from the first separation process. The effects on ABCC11-mediated [1,2,6,7-^3^H(N)]-DHEA-S transport activity were investigated by the vesicle transport assay; 1% methanol was used for the vehicle control. Data are expressed as % of vehicle and the mean ± SD; *n* = 3. **, *p* < 0.01 vs. control (Dunnett’s test).

To determine the ABCC11-inhibitory ingredients in the water extract of soybeans, further fractionation was conducted with liquid chromatographic separations in a total of three steps (Figure 3). First, the water extract was separated with a preparative MPLC system to yield 12 fractions (Fr.#1-12) (Figure 4a). The ABCC11-inhibitory activities of these 12 fractions were measured at 100 ppm (Figure 4b). Fr.#1-9 showed no significant effect, whereas Fr.#10-12 significantly inhibited the ABCC11-mediated DHEA-S transport. Secondly, since Fr.#11 exhibited the highest activity, we next further separated it with a similar preparative MPLC to give a total of six subfractions (Fr.#11-1 to Fr.#11-6), as described in Materials and Methods (Section 2.7). Monitoring the MPLC effluent at 254 nm showed that, among the six subfractions, the main compounds were collected in Fr.#11-5, which had the highest ABCC11-inhibitory activity (approximately 22% of inhibition at 50 ppm) among the six subfractions.

**Figure 5:**
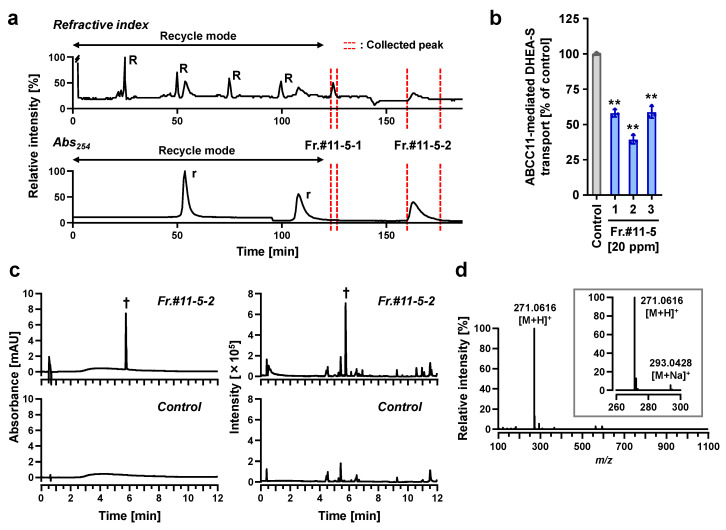
Isolation of an ABCC11-inhibitory ingredient by means of recycling preparative HPLC. (**a**) Recycling preparative HPLC chromatograms for the separation of fractions Fr.#11-5-1 and Fr.#11-5-2. The upper chromatogram was recorded with a refractive index detector, and the lower one was recorded with a diode array and multiple-wavelength detector at 254 nm. After separation under the recycling mode (0–120 min), the mode was changed; Fr.#11-5-1 (123–126 min) and Fr.#11-5-2 (160–176 min) were collected, and all the wastes were collected and further processed as Fr.#11-5-3. R, recycled peaks for Fr.#11-5-1; r, recycled peaks for Fr.#11-5-2. (**b**) ABCC11-inhibitory activities of each subfraction (20 ppm) in terms of ABCC11-mediated [1,2,6,7-^3^H(N)]-DHEA-S transport activity measured by the vesicle transport assay; 1% DMSO was used for the vehicle control. Data are expressed as % of vehicle and the mean ± SD; *n* = 3. **, *p* < 0.01 vs. control (Dunnett’s test). (**c**) Purity verification of the isolated ingredient in Fr.#11-5-2 by spectrometric analyses. Left: UV chromatograms recorded at 265 nm. Right: LC-quadrupole time-of-flight-MS (LC-Q-TOF-MS) base peak chromatograms, excluding peaks derived from the plasticizing materials and injected solvent. †, a specific peak in Fr.#11-5-2 with a retention time of 5.83 min. (**d**) Full scan mass spectrum obtained in the positive ion mode of this peak (indicated by † in **c**) at 5.83 min. The inset is the magnified view for ions at *m*/*z* 271.0616 and 293.0428, which corresponded to the [M + H]^+^ and [M + Na]^+^ of the target constituent, respectively.

Thirdly, to isolate the substances responsible for the ABCC11 inhibition, Fr.#11-5 was further subjected to recycling HPLC, which was repeated to afford components from peak #11-5-1 and peak #11-5-2 (denoted as Fr.#11-5-1 and Fr.#11-5-2, respectively; Figure 5a). All the wastes of this process were collected and further processed as Fr.#11-5-3. All three subfractions showed ABCC11-inhibitory activities at 20 ppm, and Fr.#11-5-2 was the most active (Figure 5b) and therefore the object of further analysis. Of note, the re-chromatography of Fr.#11-5-2 followed by LC-Q-TOF-MS and LC-DAD analyses suggested that this subfraction was mainly composed of a single substance that should be responsible for the ABCC11-inhibitory activity (Figure 5c). Indeed, a full LC-Q-TOF-MS scan of Fr.#11-5-2 revealed a constituent with a retention time of 5.83 min. Ions were detected in the positive ion mode at *m*/*z* 271.0616 and 293.0428, which corresponded to the [M+H]^+^ and [M+Na]^+^ of the constituent, respectively (Figure 5d).

### 3.4. Structural Characterization of the Putative ABCC11 Inhibitor Derived from Soybeans

**Figure 6:**
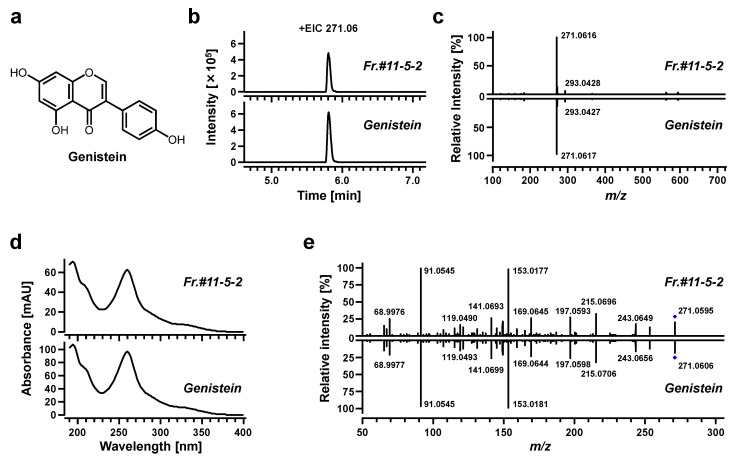
Chemical characterization of an ABCC11 inhibitory activity-guided fraction from soybean extract. Fraction (Fr.) #11-5-2 (upper panels) and authentic genistein (lower panels) were analyzed by a high-performance liquid chromatography instrument coupled with a diode array and multiple wavelength detector (DAD) and Q-TOF-MS system. (**a**) Chemical structure of genistein. (**b**) Extracted ion chromatograms (EICs) with a single peak at *m*/*z* 271.0621 in the positive ESI spectrum. (**c**) MS spectrums with a retention time of 5.83 min for the parent ion. (**d**) DAD spectrums. (**e**) Information on the fragment ions derived from MS/MS analyses.

We next conducted a series of spectrometric analyses (Figure 6) to obtain structural information about the candidate active ingredient, which was almost completely isolated from the soybean extract into Fr.#11-5-2. Based on accurate mass information from the LC-Q-TOF-MS analysis (Figure 5d), the elemental composition of the target analyte was determined as C_15_H_10_O_5_ (Δ−5.51 and Δ−2.84 ppm from [M+H]^+^ and [M+Na]^+^, respectively). The three major soy isoflavones are genistein, daidzein, and glycitein, with the respective formulas (monoisotopic mass) of C_15_H_10_O_5_ (270.0528), C_15_H_10_O_4_ (254.0579), and C_16_H_10_O_4_ (284.0685) [17]. Additionally, isoflavones exhibit an intense UV absorption between 240 and 280 nm associated with their benzoyl system, and the target analyte showed a similar spectrometric feature. Therefore, we hypothesized that the active ingredient would be genistein (Figure 6a). This hypothesis was tested by spectroscopic analyses, which demonstrated that the Fr.#11-5-2 and authentic genistein were identical in their retention time (Figure 6b), accurate mass of parent ion and the ratios of adduct ions (Figure 6c), photoabsorption spectrum (Figure 6d), and MS/MS spectrum (Figure 6e). Hence, the isolated substance should be genistein.

### 3.5. Identification of the Active Ingredient as Genistein

**Figure 7:**
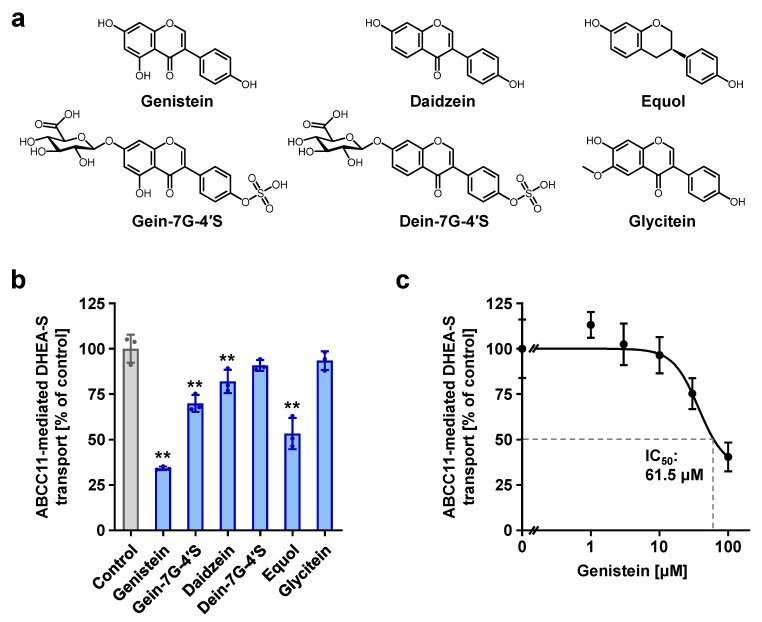
Effects of soybean flavonoids and their metabolites on the transport activity of ABCC11. (**a**) Chemical structures. Gein-7G-4’S, genistein 7-β-D-glucuronide 4’-sulfate; dein-7G-4’S, daidzein 7-β-D-glucuronide 4’-sulfate. (**b**) Inhibitory effects of each flavonoid (100 μM) on ABCC11-mediated [1,2,6,7-^3^H(N)]-DHEA-S transport. (**c**) Concentration-dependent inhibition of ABCC11-mediated DHEA-S transport by genistein. Data are expressed as % of vehicle and the mean ± SD; *n* = 3–6. **, *p* < 0.01 vs. control (Dunnett’s test).

To check whether genistein was indeed responsible for inhibiting the ABCC11 function, we examined the effects of genistein and the other two major soy isoflavones (daidzein and glycitein), as well as their metabolites (genistein 7-β-D-glucuronide 4’-sulfate, daidzein 7-β-D-glucuronide 4’-sulfate, and equol) on the ABCC11 function (Figure 7a,b). As expected, genistein inhibited ABCC11; its ABCC11-inhibitory activity was the highest among the tested compounds at 100 μM. The further examination of its concentration-dependent inhibitory effects revealed an IC_50_ of 61.5 μM (Figure 7c). If the Fr.#11-5-2 of soybean extract only contained genistein, 20 ppm of this subfraction corresponded to approximately 74 μM of genistein. The detected ABCC11-inhibitory effect of Fr.#11-5-2 at 20 ppm was approximately 40% (Figure 5b), and this is consistent with the measured concentration-dependent effects of genistein (Figure 7c). After combining these results and the determined structural characters (Figure 6), we concluded that the active ingredient in the Fr.#11-5-2 was indeed genistein.

Daidzein and glycitein only exhibited weak and minimal ABCC11-inhibitory activity, respectively. (*S*)-equol, which is a daidzein-derived metabolite produced by the intestinal bacterial flora in human intestines [18], showed a stronger effect than daidzein (Figure 7b). Moreover, compared with the non-conjugated forms, the glucuronide-sulfate diconjugates of genistein and daidzein showed lower inhibitory activities, suggesting that the polyfunctionalization-mediated structural enlargement might affect the interaction between the soy isoflavones and ABCC11 protein.

### 3.6. Investigation of ABCC11-Inhibitory Activities of Other Dietary Flavonoids

**Figure A1:**
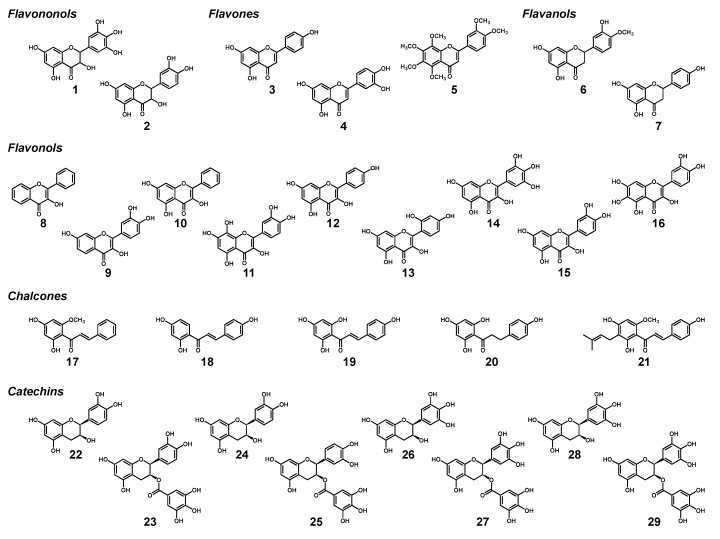
Chemical structures of dietary flavonoids tested in this study. **1**, dihydromyricetin; **2**, taxifolin; **3**, apigenin; **4**, luteolin; **5**, nobiletin; **6**, hesperetin; **7**, naringenin; **8**, 3-hydroxyflavone; **9**, fisetin; **10**, galangin; **11**, gossypetin; **12**, kaempferol; **13**, morin; **14**, myricetin; **15**, quercetin; **16**, quercetagetin; **17**, cardamonin; **18**, isoliquiritigenin; **19**, naringenin chalcone; **20**, phloretin; **21**, xanthohumol; **22**, (+)-catechin; **23**, (−)-catechin gallate; **24**, (−)-epicatechin; **25**, (−)-epicatechin gallate; **26**, (−)-epigallocatechin; **27**, (−)-epigallocatechin gallate; **28**, (+)-gallocatechin; and **29**, (−)-gallocatechin gallate.

Finally, we investigated the effects of other dietary flavonoids of interest on the ABCC11 function. The chemical structures of the selected compounds are shown in Figure A1. As shown in Table 2, at 100 μM (the same concentration used in Figure 7b), 13 of the flavonoids lowered the ABCC11-mediated DHEA-S transport to less than 30% of that of the control. Among them, luteolin, nobiletin, myricetin, quercetagetin, isoliquiritigenin, and phloretin powerfully inhibited the transport activity of ABCC11. Additionally, hardly any ABCC11-inhibitory activity was observed for (+)-catechin, (−)-epicatechin, (−)-epigallocatechin, and (+)-gallocatechin in this study, but their galloylated forms inhibited ABCC11, thus suggesting that the gallic acid esterified with catechins would be an important chemical structure for an interaction with ABCC11. These results provide a framework for the further investigation of naturally derived ABCC11 inhibitors.

## 4. Discussion

In this study, we examined the effects of water extracts of various dietary plant materials on ABCC11-mediated DHEA-S transport activity as an indicator of their ABCC11 inhibitory function (Figure 2). Among them, the extract of soybeans exhibited the strongest inhibition. Moreover, we identified genistein as an active ingredient responsible for the activity in the extract (Figure 4, Figure 5, Figure 6 and Figure 7). Hitherto, interactions between ABC proteins and phytochemicals, especially flavonoids, have attracted a lot of interest within the frameworks of multi-drug resistance (MDR) in cancer chemotherapy and the intestinal absorption of a variety of drugs, bioactive food ingredients, and/or toxins upon oral uptake because most ABC proteins are known to significantly affect the pharmacokinetic features of their substrate xenobiotics. In this way, the effects of flavonoids on MDR-related and/or intestinal ABC transporters, such as ABCB1 (also known as P-glycoprotein—P-gp), ABCG2 (breast cancer resistance protein—BCRP), and ABCC2 (multidrug resistance-associated protein 2—MRP2), have been studied, including the inhibitory effects of genistein on several ABC transporters [20,21,22,23]. However, to the best of our knowledge, no studies have examined the effects of phytochemicals on ABCC11 function. In fact, in a completely different context, the present study is the first to address and demonstrate the nutrient(s)-mediated ABCC11 inhibition by food extracts and dietary flavonoids.

Our findings may also provide a deeper understanding of the beneficial effects of flavonoids, especially soy isoflavones, that have been proposed to have a number of positive effects on human health [17,24,25]. Though the results have not been entirely consistent, there is considerable interest in using soy isoflavones to prevent cardiovascular diseases, certain types of cancer, menopausal symptoms, etc. This point of view is also supported by a recent umbrella review [26], which reported that the consumption of soy and isoflavones generally provides more benefit than harm in a series of health outcomes. While soy-based foods are traditionally consumed mainly in some Asian countries, their potential health effects have attracted growing attention from health-conscious consumers elsewhere, especially in Western countries [27]. Given this global interest, whether soy flavonoids can ameliorate the constitution causing AO or not is worth studying from the perspectives of dermatology and functional food ingredients.

Previous studies on the bioavailability and metabolism of isoflavones in humans have found that most of the circulating isoflavones are the phase II metabolites including glucuronides and sulfates [28,29,30], as well as that aglycons such as genistein and daidzein have good affinity for protein binding (>80%) [31,32]. Additionally, after the oral administration of isoflavones to humans (approximately 300 or 600 mg/day genistein and half this amount of daidzein), the plasma levels of aglycones were only in hundreds of nano molar range [33]. Hence, it will not be easy to achieve clinically relevant plasma concentrations of unbound isoflavones to inhibit ABCC11 expressed in the apocrine glands. On the other hand, given that the human axillary apocrine glands open onto the hair follicles that lead to the skin surface [34], the administration of natural extracts with ABCC11-inhibitory activity or their isolated active ingredients on the affected skin may inhibit ABCC11. For this to be effective, the treatment must produce appropriate levels of the active ingredients in the apocrine glands and also must be safe for humans. In this context, our findings here could contribute to the development of medical creams and cosmetic products targeting body odor.

Our results have also revealed a variety of dietary flavonoids that act as inhibitors for ABCC11 (Figure 7 and Table 2). However, how the structural components affect the inhibition needs to be elucidated. With isoflavones, a hydroxy group at C5 and a carbonyl group at C4 could possibly contribute positively and negatively to the ABCC11-inhibitory activities, respectively, as shown in Figure 7. On the other hand, it remains inconclusive whether the existence of a C_2_ = C_3_ double bond, a well-documented element for various bioactivities of flavonoids [35], might contribute to the inhibitory activity. To gain more insight into the relationship between the chemical structure of tested flavonoids and the inhibition of ABCC11-mediated DHEA-S transport activity, the quantitative structure–activity relationship underlying the ABCC11-flavonoids interactions should be investigated in the future.

Some of the limitations of our study and possible future directions are as follows. First, the present study was only an in vitro evaluation for the ABCC11-inhibitory activities of food ingredients. To further investigate the pathophysiological impact of our findings in the context of AO, in vivo evaluations in animals on the scale of academic laboratory are desirable. However, mice and rats have no putative orthologous gene corresponding to the human *ABCC11* [10,36]. On the other hand, previous studies have suggested that isoflavones are fairly safe for humans—exposure to them does not seem to negatively influence human health, at least at the investigated intake levels in reported cases [24,37]. Considering these facts, well-designed human studies are highly warranted.

Second, our data indicated that in addition to genistein, soybeans contain other ABCC11-inhibitory ingredients. One of them could be daidzein, although we could not isolate it from soybean extract in the present study. Regarding the fractions obtained in the first separation step (Fr.#1-12), qualitative evaluation with accurate mass chromatograms revealed that daidzein and genistein were separately fractionated into the Fr.#10 and Fr.#11, respectively. Besides Fr.#11, Fr.#10 and Fr.#12 also showed noticeable ABCC11-inhibitory activity (Figure 4b). Thus, the activity of Fr.#10 could be at least attributable to daidzein. On the other hand, judging from the UV absorption features, the ABCC11 inhibitor(s) in Fr.#12 must be non-flavonoid substances. Moreover, unknown active compounds were collected in the recycling HPLC fractions Fr.#11-5-1 and Fr.#11-5-3 (Figure 5b). Given that these fractions had little absorption peak at 254 nm (Figure 5a), such unknown compounds may not be isoflavones. The identification of these compounds and the verification of their ABCC11-inhibitory activities should be carried out in the future.

## 5. Conclusions

In conclusion, we found that the soybean extract inhibits the transport activity of ABCC11. From this extract, we successfully identified genistein, a compound known to be fairly safe for humans, as an active ingredient. Additionally, to the best of our knowledge, the present study is the first one demonstrating that some dietary flavonoids can inhibit ABCC11, at least in vitro. While human studies are needed to examine the effects of ABCC11-inhibitory phytochemicals on the AO phenotype, our findings here may provide a new clue for treating AO.

## Figures and Tables

**Table 1:** Key resources.

Reagent or Resource	Source	Identifier
***Antibodies***		
Rat monoclonal anti-MRP8 (ABCC11) antibody	Abcam, Cambridge, MA, USA	Cat# ab91452 [M8I-74]; RRID: AB_2049125
Rabbit polyclonal anti-Na^+^/K^+^-ATPase α antibody	Santa Cruz Biotechnology, Santa Cruz, CA, USA	Cat# sc-28800; RRID: AB_2290063
Goat anti-rat IgG–horseradish peroxidase (HRP) conjugate	GE Healthcare, Buckinghamshire, UK	Cat# NA935V; RRID: AB_772207
Donkey anti-rabbit IgG–HRP conjugate	GE Healthcare, Buckinghamshire, UK	Cat# NA934V; RRID: AB_772206
***Chemicals***		
Clear-sol II	Nacalai Tesque, Kyoto, Japan	Cat# 09136-83
Dehydroepiandrosterone sulfate, sodium salt, [1,2,6,7^-3^H(N)]	PerkinElmer, Waltham, MA, USA	Cat# NET860; 60.0 Ci/mmol
Dimethyl Sulfoxide	Nacalai Tesque, Kyoto, Japan	Cat# 13445-74; CAS: 67-68-5
Methanol	Nacalai Tesque, Kyoto, Japan	Cat# 21929-23; CAS: 67-56-1
3-Hydroxyflavone	Tokyo Chemical Industry, Tokyo, Japan	Cat# H0379; CAS: 577-85-5; Purity: ≥98%
Apigenin	FUJIFILM Wako Pure Chemical, Osaka, Japan	Cat# 016-18911; CAS: 520-36-5; Purity: ≥95%
Cardamonin	R&D systems, Minneapolis, MN, USA	Cat# 2509/10; CAS: 19309-14-9; Purity: ≥98%
Daidzein	FUJIFILM Wako Pure Chemical, Osaka, Japan	Cat# 043-28071; CAS: 486-66-8; Purity: ≥98%
Daidzein 7-β-D-glucuronide 4’-sulfate disodium salt	Toronto Research Chemicals, North York, ON, Canada	Cat# D103525; CAS: 1041134-19-3; Purity: N/A
Dihydromyricetin	EXTRASYNTHESE, Genay, France	Cat# 1351-10mg; CAS: 27200-12-0; Purity: ≥95%
Fisetin	LKT Labs, Minneapolis, MN, USA	Cat# F3473; CAS: 528-48-3; Purity: ≥97%
Galangin	ChromaDex, Irvine, CA, USA	Cat# ASB-00007030-010; CAS: 548-83-4; Purity: N/A
Genistein	FUJIFILM Wako Pure Chemical, Osaka, Japan	Cat# 073-05531; CAS: 446-72-0; Purity: ≥98%
Genistein 7-β-D-glucuronide 4’-sulfate disodium salt	Toronto Research Chemicals, North York, ON, Canada	Cat# G349980; CAS: 176045-29-7; Purity: N/A
Glycitein	FUJIFILM Wako Pure Chemical, Osaka, Japan	Cat# 070-04701; CAS: 40957-83-3; Purity: ≥98%
Gossypetin	ChromaDex, Irvine, CA, USA	Cat# ASB-00007390-010; CAS: 489-35-0; Purity: N/A
Hesperetin	FUJIFILM Wako Pure Chemical, Osaka, Japan	Cat# 320-93841; CAS: 520-33-2; Purity: ≥96%
Isoliquiritigenin	Tokyo Chemical Industry, Tokyo, Japan	Cat# I0822; CAS: 961-29-5; Purity: ≥97%
Kaempferol	FUJIFILM Wako Pure Chemical, Osaka, Japan	Cat# 110-00451; CAS: 520-18-3; Purity: ≥95%
Luteolin	Cayman Chemical, Ann Arbor, MI, USA	Cat# 10004161; CAS: 491-70-3; Purity: ≥98%
Morin	Combi-Blocks, San Diego, CA, USA	Cat# QC-0527; CAS: 480-16-0; Purity: ≥98%
Myricetin	FUJIFILM Wako Pure Chemical, Osaka, Japan	Cat# 137-16791; CAS: 529-44-2; Purity: ≥98%
Naringenin	Tokyo Chemical Industry, Tokyo, Japan	Cat# N0072-5g; CAS: 67604-48-2; Purity: ≥93%
Naringenin chalcone	ChromaDex, Irvine, CA, USA	Cat# ASB-00014207-005; CAS: 73692-50-9; Purity: N/A
Nobiletin	FUJIFILM Wako Pure Chemical, Osaka, Japan	Cat# 149-09341; CAS: 478-01-3; Purity: N/A
Phloretin	FUJIFILM Wako Pure Chemical, Osaka, Japan	Cat# 160-17781; CAS: 60-82-2; Purity: ≥98%
Quercetagetin	ChromaDex, Irvine, CA, USA	Cat# ASB-00017020-005; CAS: 90-18-6; Purity: N/A
Quercetin	ChromaDex, Irvine, CA, USA	Cat# ASB-00017030-010; CAS: 117-39-5: Purity: ≥97%
(*S*)-Equol	Cayman Chemical, Ann Arbor, MI, USA	Cat# 10010173; CAS: 531-95-3; Purity: ≥98%
Taxifolin	EXTRASYNTHESE, Genay, France	Cat# 1036; CAS: 17654-26-1; Purity: N/A
Xanthohumol	TOKIWA PHYTOCHEMICAL, Chiba, Japan	Cat# P2217; CAS: 569-83-5; Purity: ≥98%
(+)-Catechin	FUJIFILM Wako Pure Chemical, Osaka, Japan	Cat# 038-23461; CAS: 154-23-4; Purity: ≥99%
(−)-Catechin gallate	Nagara Science, Gifu, Japan	Cat# NH021302; CAS: 130405-40-2; Purity: ≥98%
(−)-Epicatechin	FUJIFILM Wako Pure Chemical, Osaka, Japan	Cat# 059-06751; CAS: 490-46-0; Purity: ≥98%
(−)-Epicatechin gallate	FUJIFILM Wako Pure Chemical, Osaka, Japan	Cat# 052-06741; CAS: 1257-08-5; Purity: ≥98%
(−)-Epigallocatechin	FUJIFILM Wako Pure Chemical, Osaka, Japan	Cat# 059-08951; CAS: 970-74-1; Purity: ≥99%
(−)-Epigallocatechin gallate	FUJIFILM Wako Pure Chemical, Osaka, Japan	Cat# 056-08961; CAS: 989-51-5; Purity: ≥99%
(+)-Gallocatechin	FUJIFILM Wako Pure Chemical, Osaka, Japan	Cat# 075-06331; CAS: 970-73-0; Purity: ≥99%
(−)-Gallocatechin gallate	Nagara Science, Gifu, Japan	Cat# NH021402; CAS: 4233-96-9; Purity: ≥98%
***Adenoviruses***		
ABCC11-expressing adenovirus	Toyoda et al. 2017 [9]	N/A
EGFP-expressing adenovirus	Toyoda et al. 2017 [9]	N/A
***Recombinant DNA***		
The complete human ABCC11 cDNA in pcDNA3.1/hyg(−)	Toyoda et al. 2009 [4]	NCBI Reference Sequence: NM_033151
***Experimental Models: Cell Lines***		
293A	Invitrogen, Waltham, MA, USA	R70507

N/A, not available.

**Table 2:** ABCC11-inhibitory activities of dietary flavonoids.

Class	Tested Food Ingredients	ABCC11-Mediated DHEA-S Transport (% of Control)	*p* Value †
Flavanonol	Dihydromyricetin	56.9 ± 23.4	0.043
	Taxifolin	43.2 ± 26.0	0.032
Flavone	Apigenin	25.3 ± 12.5	0.005
	Luteolin	0 *	<0.001
	Nobiletin	0 *	<0.001
Flavanone	Hesperetin	5.2 ± 26.9	0.013
	Naringenin	38.4 ± 31.6	0.039
Flavonol	3-Hydroxyflavone	91.7 ± 1.1	0.003
	Fisetin	37.7 ± 1.0	<0.001
	Galangin	63.6 ± 5.9	0.004
	Gossypetin	46.0 ± 8.4	0.004
	Kaempferol	42.8 ± 1.0	<0.001
	Morin	41.5 ± 3.4	<0.001
	Myricetin	0 *	0.002
	Quercetin	34.6 ± 18.3	0.013
	Quercetagetin	0 *	<0.001
Chalcone	Cardamonin	73.4 ± 10.8	0.025
	Isoliquiritigenin	0 *	<0.001
	Naringenin chalcone	12.4 ± 4.2	<0.001
	Phloretin	0.6 ± 3.9	<0.001
	Xanthohumol	19.7 ± 13.6	0.005
Catechins	(+)-Catechin	91.9 ± 5.9	0.071 (NS)
	(−)-Catechin gallate	10.5 ± 6.0	0.001
	(−)-Epicatechin	85.2 ± 24.4	0.202 (NS)
	(−)-Epicatechin gallate	29.8 ± 6.8	0.002
	(−)-Epigallocatechin	83.6 ± 29.0	0.215 (NS)
	(−)-Epigallocatechin gallate	37.5 ± 7.0	0.002
	(+)-Gallocatechin	87.6 ± 27.6	0.259 (NS)
	(−)-Gallocatechin gallate	24.0 ± 2.5	<0.001

Inhibitory effects of each food ingredient (100 μM) on ABCC11-mediated [1,2,6,7-^3^H(N)]-DHEA-S transport activity were investigated by using plasma membrane vesicles (0.5 mg/mL in the reaction mixture) prepared form ABCC11-expressing or control adenovirus-infected 293A cells. Additionally, major green tea catechins, based on a previous study [19], were tested in this study. Data are expressed as % of vehicle and the mean ± SD; *n* = 3. *, Values were calculated under 0; †, one-sample *t*-test (vs. vehicle control as 100%); NS, not significantly different from control (*p* > 0.05).

## Appendix A

**Table A1:** Tested plant materials.

Descriptions in this Study	Common Names	Academic Names	Details of Material *
*Arctium lappa* root	Burdock root tea	*Arctium lappa*	Dried root for tea
*Aspalathus linearis* leaves	Rooibos tea leaves	*Aspalathus linearis*	Dried leaves for tea
Barley (*Hordeum vulgare*) miso	Barley miso	*Hordeum vulgare* ^#^	Japanese traditional fermented product
*Citrus aurantiifolia* peel	Lime	*Citrus aurantiifolia*	Peel
*Citrus depressa* peel	Shikuwasa	*Citrus depressa*	Peel
*Citrus junos* peel	Yuzu	*Citrus junos*	Peel
*Citrus maxima* peel	Pomelo	*Citrus maxima*	Peel
*Citrus maxima* placenta	Pomelo	*Citrus maxima*	Inner white and soft tissue layer
*Citrus natsudaidai* peel	Suruga elegant	*Citrus natsudaidai*	Peel
*Citrus reticulata var poonensis* peel	Ponkan	*Citrus reticulata var. poonensis*	Peel
*Citrus sinensis* peel	Blood orange	*Citrus sinensis*	Peel
*Citrus sphaerocarpa* peel	Kabosu	*Citrus sphaerocarpa*	Peel
*Citrus sudachi* peel	Sudachi	*Citrus sudachi*	Peel
(*Citrus unshiu × Citrus sinensis*) *× Citrus reticulata* peel	Siranuhi (Dekopon)	*(Citrus unshiu × Citrus sinensis) × Citrus reticulata*	Peel
*Citrus × Paradisi* peel	Grapefruit	*Citrus × Paradisi*	Peel
*Citrus × Tangelo* peel	Mineola orange (Tangelo)	*Citrus × Tangelo*	Peel
Commercially available lemon juice A	Not available	Not available	Commercially available product ^‡^
Commercially available lemon juice B	Not available	Not available	Commercially available product ^‡^
Commercially available lemon juice C	Not available	Not available	Commercially available product ^‡^
*Ditrus sinensis Osbeck var. brasiliensis Tanaka* peel	Navel orange	*Ditrus sinensis Osbeck var. brasiliensis Tanaka*	Peel
*Fortunella* peel	Kumquat	*Fortunella*	Peel
*Glycine max*	Soybeans(yellow soybean)	*Glycine max*	Dried product
Hidenmame (*Glycine max*)	Soybeans(green soybean)	*Glycine max*	Dried product
*Humulus lupulus* L. cone	Hop	*Humulus lupulus* L.	Frozen hop cone
*Ilex paraguariensis* leaves (roasted)	Yerba mate tea leaves	*Ilex paraguariensis*	Dried and roasted leaves for tea
*Jasminum polyanthum* leaves	Jasmine tea leaves	*Jasminum polyanthum*	Dried leaves for tea
*Matricaria recutita*	Chamomile	*Matricaria recutita*	Dried herb product
*Prunus domestica* extract	Prune extract	*Prunus domestica*	Product of prune pulp extract ^‡^
*Prunus domestica* pulp	Prune	*Prunus domestica*	Product of prune pulp without seed
*Psidium guajava* leaves (from China)	Guava tea leaves	*Psidium guajava*	Dried leaves for tea cultivated in China
*Psidium guajava* leaves (from Japan)	Guava tea leaves	*Psidium guajava*	Dried leaves for tea cultivated in Japan
Rice (*Oryza sativa*) miso	Rice miso	*Oryza sativa* ^#^	Japanese traditional fermented product
*Smallanthus sonchifolius* tea powder	Yacon tea powder	*Smallanthus sonchifolius*	Dried product
Soybean (*Glycine max*) miso	Soybean miso	*Glycine max* ^#^	Japanese traditional fermented product

*, Unless otherwise indicated, fresh materials were used; ^#^, academic name of main material of miso product; ^‡^, after defatting via liquid-liquid partition with an equal volume of ethyl acetate, the obtained water phase of each juice or extract was subjected to lyophilization.
